# Airborne-Platform-Assisted Transmission and Control Separation for Multiple Access in Integrated Satellite–Terrestrial Networks

**DOI:** 10.3390/s25154732

**Published:** 2025-07-31

**Authors:** Chaoran Huang, Xiao Ma, Xiangren Xin, Weijia Han, Yanjie Dong

**Affiliations:** 1 School of Physics and Information Technology, Shaanxi Normal University, Xi’an 710119, China; chuang@snnu.edu.cn (C.H.); wjhan@snnu.edu.cn (W.H.); 2 Information Construction and Management Department, Shaanxi Normal University, Xi’an 710119, China; 3 Artificial Intelligence Research Institute, Shenzhen MSU-BIT University, Shenzhen 518172, China; ydong@smbu.edu.cn

**Keywords:** space–air–ground networks, separation of transmission and control, satellite communications

## Abstract

Currently, the primary random access protocol for satellite communications is Irregular Repetition Slotted ALOHA (IRSA). This protocol leverages interference cancellation and burst repetition based on probabilistic distributions, achieving up to 80% channel utilization in practical use. However, it faces three significant issues: (1) low channel utilization with smaller frame sizes; (2) drastic performance degradation under heavy load, where channel utilization can be lower than that of traditional Slotted ALOHA; and (3) even under optimal load and frame sizes, up to 20% of the valuable satellite channel resources are still wasted despite reaching up to 80% channel utilization. In this paper, we propose the Separated Transmission and Control ALOHA (STCA) protocol, which introduces a space–air–ground layered network and separates the access control process from the satellite to an airborne platform, thus preventing collisions in satellite channels. Additionally, the airborne-platform estimates the load to ensure maximum access rates. Simulation results demonstrate that the STCA protocol significantly outperforms the IRSA protocol in terms of channel utilization.

## 1. Introduction

With the advancement of 6G technology, more and more devices are supporting satellite communications [1]. It is foreseeable that a large amount of Satellite User Equipment (SUE) will contend for scarce satellite resources, while the existing multiple access protocol Irregular Repetition Slotted ALOHA (IRSA) faces communication bottlenecks due to load and frame size issues. Therefore, a new multiple access protocol is needed to support the increasing amount of SUE in satellite communications.

In the context of wireless communications, multiple access protocols include fixed multiple access and random multiple access [2]. However, fixed multiple access protocols cannot be applied to satellite communications for the following reasons: (1) the frequent changes in the amount of SUE and (2) the bursty traffic demand from SUE. Therefore, random multiple access has become a major research topic in satellite communications. The earliest application of random multiple access can be traced back to the ALOHA protocol. With the advancement of radio frequency instruments, Carrier Sense Multiple Access (CSMA) was introduced, which further improved network throughput compared with ALOHA and its variants [3]. However, in the CSMA protocol, each piece of SUE cannot monitor neighboring SUE and becomes a hidden terminal to them. Additionally, due to the long-distance propagation between satellites and SUE, each piece of SUE needs to listen for a long time before transmission. Therefore, CSMA becomes ineffective in satellite communications. By considering the listening duration and the random transmission of the vanilla ALOHA protocol, future multiple access protocols for satellite communications are expected to be based on Slotted ALOHA [4].

The Slotted ALOHA (SA) protocol is a classic variant of ALOHA and is widely used in wireless networks. It discretizes time, resulting in a lower collision probability compared with the ALOHA protocol. In the field of satellite communications, channel utilization of satellite devices is of great interest. In [5], the Diversity Slotted ALOHA (DSA) protocol, which slightly improved the channel utilization of SA by introducing burst repetition under low-load conditions, was proposed. In [6], Contention Resolution Diversity Slotted ALOHA (CRDSA), which further improved system channel utilization by introducing interference cancellation techniques, was proposed. Building on this, [7] proposed IRSA, which made the number of burst repetitions random, increasing the theoretical maximum channel utilization to 97%, with an actual utilization of 80%, while SA achieves only 37%.

Although the IRSA algorithm significantly improves channel utilization, it still has three unresolved issues. First, the frame size: The maximum channel utilization of the IRSA algorithm is proportional to the frame size. The more slots a frame contains, the higher the channel utilization. For example, achieving 80% channel utilization requires a frame to contain about 1000 slots for access, resulting in excessively long frame times. Additionally, the performance of the IRSA algorithm degrades significantly under high channel load, with channel utilization even lower than Slotted ALOHA. Finally, the IRSA algorithm still results in 20% channel resource waste in practical use.

It is worth noting that time delay issues caused by long-distance propagation in satellite communications further exacerbate the performance bottlenecks of existing protocols. Studies on spacecraft control systems have shown that unbounded input time delay can severely affect dynamic response stability. The integrated predictor–observer feedback control strategy, which provides important insights for handling communication time delays by synchronously estimating system states and disturbances and incorporating a predictor to compensate for time delays, was proposed in [8].

To address the above issues, this paper proposes the Separated Transmission and Control IRSA (STCA) protocol. This protocol separates transmission and access control functions, deploying the access control process on airborne access control nodes (ACNs) to form an integrated space–air–ground network.

Specifically, SUE first accesses ACNs closer to the ground and, after successful access, directly transmits information to the satellite. In this way, collisions only occur at ACNs, thereby maximizing satellite channel utilization. By offloading access control to ACNs close to the ground, the access interaction distance for SUE is shortened, reducing the transmission delay of control signaling; meanwhile, the load estimation and control algorithms mitigate the impact of load fluctuations caused by time delays by dynamically adjusting access probabilities, which echoes the idea in [8] that “time delay compensation should be combined with dynamic adjustments”. In addition, due to the separation of transmission and access control processes, access data packets contain only a small amount of information, and the access process uses minislots as access slots, which addresses the issue of excessively long frame times in IRSA caused by the need for a large number of slots. Simulation results show that the proposed STCA outperforms IRSA in terms of channel utilization.

It is worth noting that, due to the improvement in channel utilization, the retransmission delay after collisions is significantly shortened. The delay introduced by adding ACNs is much smaller than the retransmission delay; therefore, the overall delay will not increase.

The main contributions of this paper are as follows:

1. We propose the Separation of Transmission and Control ALOHA (STCA) protocol, which moves access control from the satellite to airborne access control nodes (ACNs), confining collisions to the ACN layer and improving satellite channel utilization.
2. A load estimation and control algorithm is designed to dynamically adjust the access probability of each piece of SUE based on IRSA, preventing performance degradation under high load.
3. By separating access control from data transmission, access packets contain only essential information. The use of minislots reduces frame length compared with IRSA, which requires many slots.
4. Simulation results show that STCA achieves significantly higher channel utilization and throughput stability than IRSA and traditional Slotted ALOHA, providing an efficient solution for multi-user access in satellite communications.

The rest of this paper is organized as follows: Section 2 describes the system model, including the ISAG network architecture and three spectra; Section 3 formulates the problem and optimization objectives for systems with/without SIC and variable frame length; Section 4 details the STCA protocol and its three variants; Section 5 presents numerical results comparing STCA with existing protocols; Section 6 concludes the paper.

## 2. System Model

This paper refers to the space–air–ground integrated network architecture, as discussed in [9], which addresses random access congestion issues for user equipment in massive IoT networks within such a network framework. The network studied in this work is divided into three layers: the satellite layer, the access control node layer (ACN layer), and the satellite user equipment layer (SUE layer). Based on this framework, we construct an integrated space–air–ground (ISAG) network by introducing airborne devices into the traditional satellite communication system. This ISAG network utilizes three types of spectrum to connect devices across different layers. The names and functions of these spectra are described as follows:

(1) The Contention Access Spectrum (CAS) connects the ACNs to SUE and is used for contending access and broadcasting the indices of traffic transmission allocated by the satellite;
(2) The Resource Allocation Spectrum (RAS) connects satellites to the ACNs and is used for allocating traffic transmission resources to ACNs according to the load of each ACN;
(3) The Traffic Transmission Spectrum (TTS) connects the satellites to SUE and is used to convey the traffic data within the traffic transmission resources that the SUE has competed for from the ACNs.

Using these three types of spectra, we can separate the transmission plane from the control plane.

**Figure 1:**
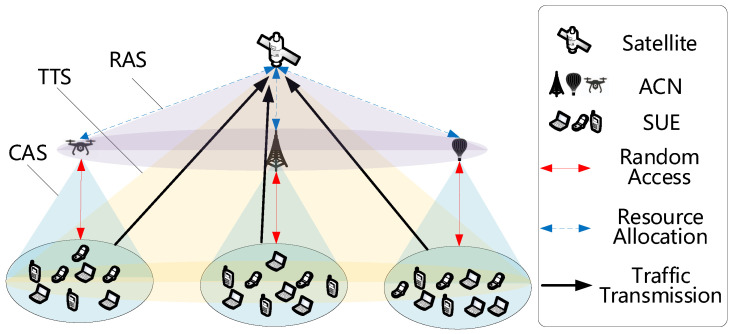
System model.

As shown in Figure 1, the considered ISAG network consists of one satellite, *J* ACNs, and several pieces of SUE. ACNs can take various forms, such as unmanned aerial vehicles, high-altitude platforms, airplanes, etc., and each ACN needs to have a base station.

The base station manages SUE access and processes access results, establishing connections with SUE by receiving its contention packets, determining access status (success, collision, or idle), and feeding back results.

Each ACN *j* is associated with $M_{j}$ pieces of SUE. For ACN *j*, a subset of SUE $M_{i,j}$ has incoming traffic packets in frame *i* ($M_{i,j}⩽M_{j}$). SUE must obtain permission to transmit. Since each ACN operates independently without communicating with others, the subsequent content will be described using one ACN as an example, without using the ACN index *j*.

**Figure 2:**
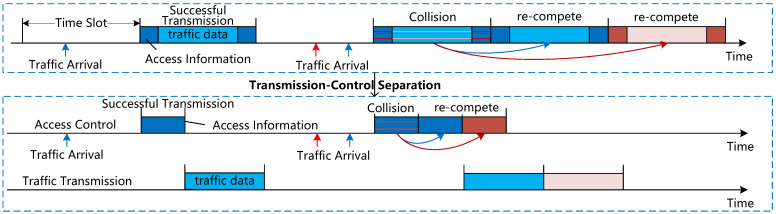
Schematic diagram of transmission–control separation architecture.

The random multiple access procedures for systems with and without transmission and control separation are illustrated in Figure 2. As depicted, when a service request arrives at a slot, it defers transmission until the beginning of the next slot to avoid collisions. A transmission is deemed successful if only one service request occupies a slot. If two or more requests attempt simultaneous transmission, a collision occurs, triggering a retransmission process after a random backoff period.

Prior to transmission and control separation, each data packet has encapsulated both service payload and access control information. Consequently, collisions result in the loss of both components. Following separation, access control information is confined to the access phase, while service payloads are transmitted exclusively in the dedicated transmission phase. Collisions are thus restricted to the access phase, enabling collision-free transmission of service data over the satellite channel. This architecture maximizes channel utilization by eliminating contention during payload transmission. This procedure is formalized in the Algorithm 1.
**Algorithm 1:** Transmission–control separation algorithm
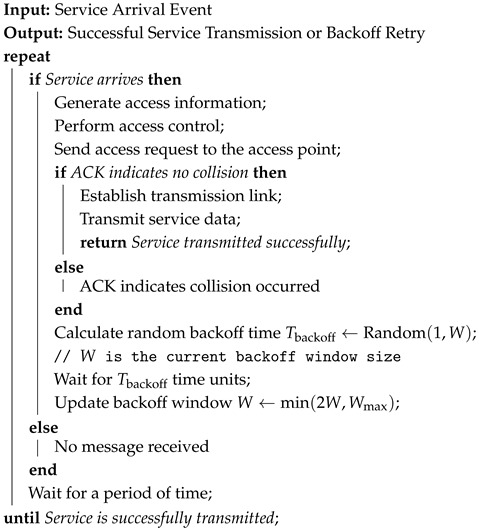


**Figure 3:**
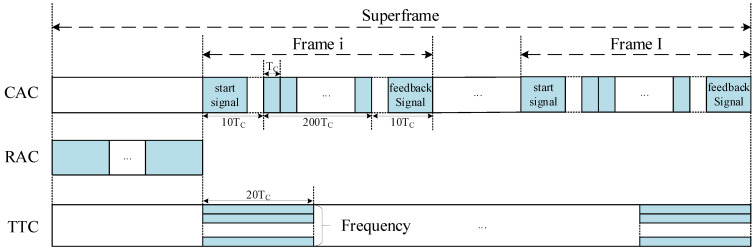
Frame structure.

As shown in Figure 3, these three types of spectra operate in a discrete-time mode and are composed of superframes, frames, slots, and minislots. The RAS only broadcasts the allocated frequency resources for each ACN at the beginning of the superframe. Since the CAS only needs to exchange identity information and the number of packets on the TTS, the duration of each CAS slot can be much shorter than the TTS slots; therefore, the frames of the CAS are composed of minislots. Within one superframe, the CAS contains *I* frames, each consisting of three parts: the first part is for broadcasting access information; the second part is for contention access, consisting of $N_{C}$ minislots; the third part is for feedback of access results. The TTS contains $N_{T}$ slots.

More specifically, each CAS slot lasts $T_{C}$ milliseconds, each RAS slot lasts $T_{A}$ milliseconds, and each TTS slot lasts $T_{T}$ ($T_{T}>T_{C}$) milliseconds. Hereafter, CAS slots will be referred to as minislots. To improve utilization efficiency, the TTS is also divided into multiple sub-channels, and each ACN has multiple sub-channels. During the uplink transmission of the CAS, each piece of SUE will randomly select several minislots at the beginning of frame *i* and transmit a contention packet over the chosen minislot. In the considered ISAG network, we assume that the SUE of different ACNs can use orthogonal CAS to contend for TTS slots. Since each minislot can support one contention packet, the contention result for each minislot can be success, collision, or idle:

- Success indicates that one single piece of SUE transmits a contention packet over the CAS slot. The successful SUE transmits the traffic packet over the TTS slots.
- Collision indicates that more than one piece of SUE transmits contention packets over the CAS slot. The collided pieces of SUE need to re-compete for another CAS slot in the next frame and store the traffic packets until no collision occurs over the chosen CAS slot.
- Idle indicates that no SUE transmits contention packet over the CAS slot.

Assume that the arrival of traffic packets from SUE is an independent Poisson process. The total arrival rate of pieces of SUE controlled by an ACN in frame *i* is denoted by $\lambda_{i}$. To control the number of pieces of SUE gaining access in each frame, each piece of SUE has a probability $P_{i}$ of being allowed to access in frame *i*. *m* represents the number of pieces of SUE contending for access, and *n* represents the number of resources being contended for. The access load of an ACN in frame *i* is defined as

(1) $$G_{i}=\frac{m}{n}=\frac{M_{i}P_{i}}{N_{C}}.$$

## 3. Problem Description

The objective of this paper is to optimize the utilization of TTS. Since random access is not performed in TTS, no collision slots occur. Moreover, as the throughput *S* of CAS per unit time increases, the probability of idle slots in TTS decreases, leading to increased channel utilization. Therefore, the optimization goal is transformed into maximizing the throughput *S* of CAS per unit time. In a random competition scenario, the throughput *S* of the channel per unit time and the channel load *G* follow the following equation:

(2) $$S=Ge^{-G}.$$

This paper considers two scenarios: one in which the ACN can only support random competition and another in which the ACN supports Serial Interference Cancellation (SIC) capability.

**Figure 4:**
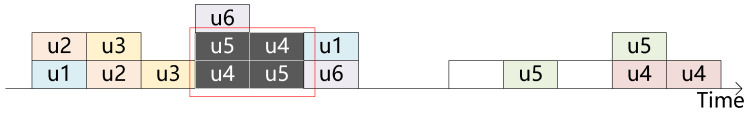
Flowchart of Successive Interference Cancellation (SIC).

The Successive Interference Cancellation (SIC) algorithm effectively enhances the capacity and spectral efficiency of multi-user systems by progressively eliminating interference in received signals. In SIC, each ground terminal generates multiple copies of each burst packet, which are transmitted in arbitrary slots of a frame. As shown in Figure 4, u1–u6 represent the burst copies of six users. Notably, only the third slot in the first frame contains a single burst (u3), referred to as a “clean slot” in this paper. All other slots contain two or more packets, which would be considered collisions in the Slotted ALOHA protocol—resulting in packet discarding and slot waste.

With SIC, u3 can be decoded first. By identifying the other copy of u3 in slot two, Successive Interference Cancellation eliminates the u3 signal from slot two, converting it into a clean slot and recovering u2. Using the recovered u2 signal, the u1 signal in slot one can then be extracted. This process continues iteratively until all decodable signals are recovered. Users u4 and u5, however, remain mutually overlapped and deadlocked, requiring retransmission in the next frame. The retransmission here can refer to the distributed queueing method in [10] and the distributed queuing random access protocol in [11], which can further improve system performance. Similar to the approach in [12], where distributed queueing was introduced into the LoRa network, a similar method could be considered here. Considering that the increase in complexity would further increase the delay, this paper employs a random backoff mechanism for retransmissions instead.

Compared with Slotted ALOHA, this approach reutilizes many burst packets that would otherwise be discarded, significantly improving the channel utilization of satellite communication networks and reducing latency. This process is termed SIC; however, its high computational complexity mandates that access control nodes possess robust processing capabilities. For systems that do not use the SIC algorithm, the optimization goal is

(3) $$P1:maxS=max\left(\frac{M_{i}P_{i}}{N_{C}}e^{-\frac{M_{i}P_{i}}{N_{C}}}\right).$$

In systems where the SIC algorithm can be used, each piece of SUE generates *l* copies during access and randomly transmits these *l* copies in $N_{C}$ minislots. The probability distribution of *l* is expressed as follows:

(4) $$\Lambda \left(x\right)=\Lambda_{1}x+\Lambda_{2}x^{2}+\ldots +\Lambda_{N_{C}}x^{N_{C}},$$

where $\Lambda_{l}$ is the probability that the SUE generates *l* copies. Each copy contains a pointer to the locations of the other copies. When a minislot is in a successful state, the pointer of the copy finds the locations of the other copies selected by the SUE. Then, the SIC algorithm is used to recover the user’s data packet. This method can convert a large number of collided minislots into successful minislots. Figure 4 is a schematic diagram of the SIC decoding process. The gray squares represent successful minislots, the white squares represent collided minislots, the gray circles indicate that the SUE has successfully gained access, and the white circles indicate that the SUE has not gained access.

In [7], the SIC algorithm has an access load threshold $G^{*}$. When *G* exceeds $G^{*}$, the normalized throughput rapidly decreases. In this paper, the higher the normalized throughput of the CAS, the higher the channel utilization rate of TTS (the more SUE successfully gains access within a unit time, the more data the satellite receives). Therefore, for systems that use the SIC algorithm, the optimization goal is

(5) $$P2:min\left(|G-G^{*}|\right)=min\left(\left|\frac{M_{i}P_{i}}{N_{C}}-G^{*}\right|\right).$$

Considering a potential scenario where a piece of SUE may be persistently randomized into a nontransmitting state due to the access probability, resulting in prolonged waiting times for some pieces of SUE, we further propose an alternative algorithm. Since the access load represents the number of accessing pieces of SUE per frame, we no longer regulate the number of accessing pieces of SUE through probability adjustment but instead control the access load by dynamically adjusting the frame length. The load expression is given as follows:

(6) $$G_{i}=\frac{m}{n}=\frac{M_{i}}{K_{i}},$$

where $K_{i}$ denotes the adjusted number of timeslots in frame *i*. The optimization objective of the method is as follows:

(7) $$P3:min\left(|G-G^{*}|\right)=min\left(\left|\frac{M_{i}}{K_{i}}-G^{*}\right|\right).$$

The three approaches mentioned above are named STCA without SIC, STCA with SIC, and STCA with variable frame length, respectively.

## 4. The Proposed STCA Protocol

Since whether the ACN has SIC capability affects the system’s optimization goal, this section introduces the operation process of two systems separately. In addition, considering the need for fairness in SUE, this section also devises an approach which is called STCA with variable frame length.

### 4.1. STCA Without SIC

First, this protocol considers three types of channels, which consist of superframes, frames, timeslots, and minislots. A competition access channel within a superframe contains *I* frames, and each frame is composed of three parts. The first part is the broadcast access information, which includes $N_{m}$ minislots and one idle minislot used for waiting for transmission. The second part is the competition access, consisting of $N_{C}$ minislots. The third part is the feedback access result, which also includes $N_{m}$ minislots and one idle minislot. The service transmission channel contains $N_{T}$ timeslots.

More specifically, each minislot in the competition access channel lasts for $T_{C}$ milliseconds, and each timeslot in the resource allocation channel lasts for $XT_{C}$ milliseconds. The duration of this timeslot can be freely set, with minimal impact on the system. Each timeslot in the service transmission channel lasts $XT_{C}$ milliseconds.

To improve utilization efficiency, the service transmission channel is further divided into multiple sub-channels, with each air access control node allocated *Y* sub-channels. During the uplink transmission in the competition access channel, each ground terminal randomly selects a minislot at the beginning of frame *i* and sends a competition packet in the selected minislot. In the considered air–ground network, it is assumed that ground terminals of different air access control nodes can use orthogonal competition access channels to contend for the service transmission channel slots. Each minislot can have three states: successful, collided, and idle.

The general process of the system is as follows: First, when the system is started for the first time, the satellite allocates service resources to the air access control nodes. Upon receiving the service channel resource allocation information, the air access control node broadcasts the service transmission channel index, competition access channel index, and access probability $P_{i}$ to the ground terminals under its control. The ground terminals contend for access to the air access control node based on these indices and the probability $P_{i}$.

Subsequently, the air access control node performs decoding and provides feedback on the access results and the access probability for the next frame to the ground terminals. The ground terminals that have successfully gained access send data to the satellite through the service transmission channel based on the feedback information. The ground terminals that have failed to gain access wait for the next frame of the competition access channel to contend for access again.

**Figure 5:**
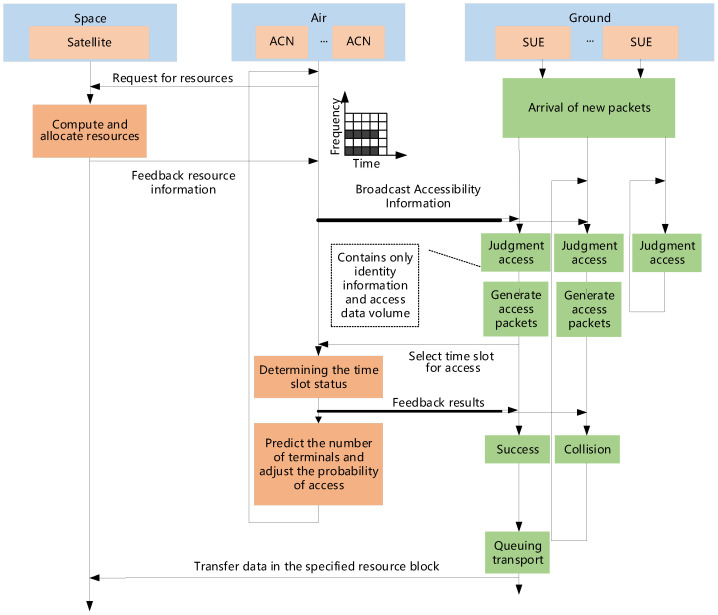
Flowchart of system without SIC.

After one superframe of operation, the air access control node sends the estimated traffic load to the satellite, and the satellite allocates the service channel resources based on the load size. The flowchart is shown in Figure 5.

The access process occurs between the ground terminals and the air access control nodes. When a data packet arrives at a ground terminal, this terminal must wait for the broadcast from the air access control node. The broadcast from the air access control node includes the access probability $P_{i}$, and this ground terminal has a probability of $P_{i}$ to send an access request. If it does not send the request, it will wait for the next frame. If it sends the request, it will select a minislot from the minislot indices broadcast by the air access control node to send the access information. Subsequently, the ground terminal waits for feedback from the air access control node, which includes the access result and the minislot in the service transmission channel where the data packet will be sent. If the ground terminal has a new data packet arriving during the access process, it will request access while sending the data packet.

Additionally, due to the difference in the duration between minislots and timeslots, when a traffic data packet in the service transmission channel is completed, several ground terminals will have already completed their access in the competition access channel. A transmission queue will be generated in the service transmission channel. When the normalized throughput of the access process is sufficient, continuous collision-free data packets will be sent to the satellite, and the channel utilization will always be 100%. The access probability $P_{i}$ can be adjusted according to the access load to ensure maximum normalized throughput. The following will detail how to predict and estimate the number of ground terminals needing access in each frame and how to adjust the access probability $P_{i}$ based on this estimate.

For the air access control node, the real access load is unknown. Therefore, it needs to estimate how many ground terminals have data packets to send in the current frame. In discrete-time channels, the access load is proportional to the number of idle minislots. The probability of a minislot being idle is

(8) $$P_{idle}={\left(\frac{N_{C}-1}{N_{C}}\right)}^{M_{i}P_{i}}.$$

Thus, the estimated number of idle minislots in a competition access channel frame can be expressed as

(9) $$n_{idle}=N_{C}P_{idle}=f\left(M_{i}\right).$$

For the air access control node, the only unknown is $M_{i}$. Thus, this expression can be written as a function $f(M_{i})$. Additionally, its inverse function can be written as

(10) $$M_{i}=f^{-1}\left(n_{idle}\right).$$

At the end of each frame, the air access control node can determine how many minislots are in the idle and successful states, which allows one to estimate how many ground terminals have data packets to send in the next frame:

(11) $$M_{i+1}=\left(M_{i}-M_{succ}\right)+\left(M-M_{i}+M_{succ}\right)\left(1-e^{-N_{C}T_{C}\lambda_{i}}\right),$$

where $M_{succ}$ is the number of ground terminals that successfully gained access in frame *i*. Since the competition access information contains identity information, $M_{succ}$ is known to the air access control node. The above equation consists of two parts: one part corresponds to the ground terminals that failed to access due to a probability of $1-P_{i}$ and need to retransmit, while the other part corresponds to those that have just reached the need to access. $\lambda_{i}$ can be iteratively estimated as

(12) $$\lambda_{i}=-\alpha \frac{\ln\left(\frac{M_{i}-M}{M_{i-1}-M_{succ}^{\prime }-M}\right)}{N_{C}T_{C}}+\left(1-\alpha \right)\lambda_{i-1},$$

where α is a constant. Since the arrival rate does not change drastically in the short term, a portion of the previous frame’s arrival rate is retained. $M_{succ}^{\prime }$ is the number of successfully accessed ground terminals in the previous frame. Combined with Equation (1), the air access control node can set the access probability $P_{i+1}$ for the next frame based on the predicted $M_{i+1}$, in order to make G=0.368, thereby maximizing the throughput per unit time.

The above method maximizes throughput by controlling the access probability. However, considering that some ground terminals may never successfully access the system, leading to fairness issues, this paper also proposes an algorithm to adjust the frame length.

### 4.2. STCA with SIC

**Figure 6:**
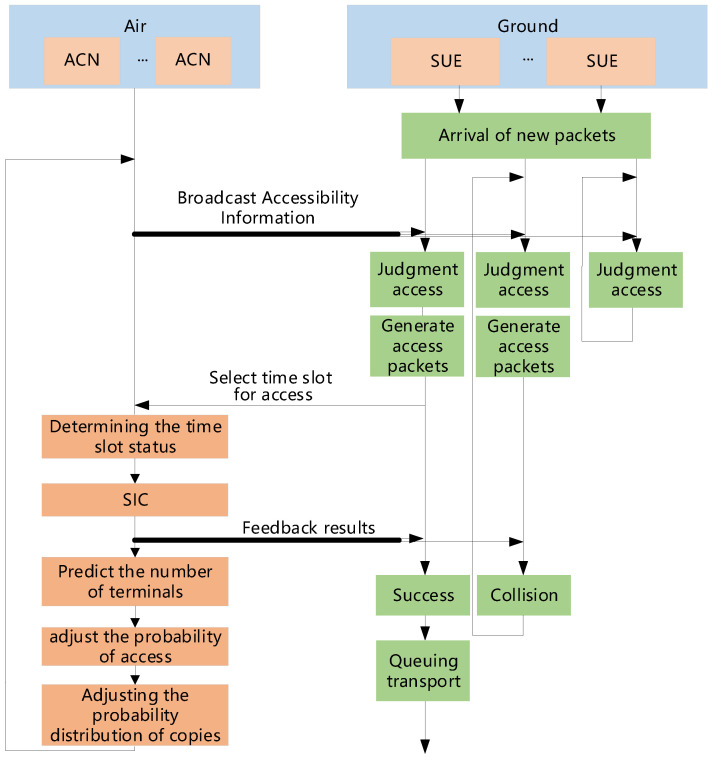
System flowchart of STCA with SIC.

The following section introduces the process of a system with SIC capability, and Figure 6 shows the system flowchart. First, when the system is started for the first time, the satellite evenly allocates the number of sub-channels to each air access control node. Upon receiving the channel resource allocation information, the air access control node broadcasts the service transmission channel index, competition access channel index, and access probability $P_{i}$ to the ground terminals under its control. The ground terminals contend for access to the air access control node based on these indices and the probability $P_{i}$. Subsequently, the air access control node performs Serial Interference Cancellation (SIC) decoding and provides feedback on the access results and the access probability for the next frame to the ground terminals. The successfully accessed ground terminals send data to the satellite through the service transmission channel based on the feedback information. The ground terminals that fail to access will wait for the next frame of the competition access channel to contend for access again. After one superframe of operation, the air access control node sends the estimated traffic load to the satellite, and the satellite allocates the number of sub-channels proportionally based on the load.

The access process occurs between the ground terminals and the air access control nodes. When a data packet arrives at a ground terminal, the terminal must wait for the broadcast from the air access control node. The broadcast from the air access control node includes the access probability $P_{i}$, and this ground terminal has a probability of $P_{i}$ to send an access request. If it does not send the request, it will wait for the next frame. If it sends the request, it will randomly generate *l* copies and select *l* minislots from the minislot indices broadcast by the air access control node to send the access information. Subsequently, the terminal waits for feedback from the air access control node, which includes the access result and the minislot in the service transmission channel where the data packet will be sent. If a new data packet arrives at the ground terminal during the access process, it will request access while sending the data packet.

Additionally, due to the difference in the duration between minislots and timeslots, when a traffic data packet in the service transmission channel is completed, several ground terminals will have already completed their access in the competition access channel. A transmission queue will be generated in the service transmission channel. When the normalized throughput of the access process is sufficient, the channel utilization will always be 100%. The access probability $P_{i}$ can be adjusted according to the access load to ensure maximum normalized throughput.

The air access control node is unaware of the actual access load. Therefore, it needs to estimate how many pieces of SUE have data packets to send in the current frame. In discrete-time channels, the access load is proportional to the number of idle minislots. The probability of a minislot being idle is

(13) Pidle=MiPi∑l=1NCrlNC−1lNClMiPi.

Thus, the estimated number of idle minislots in a frame of the CAS is

(14) $$n_{idle}=N_{C}P_{idle}=f\left(M_{i}\right).$$

For the ACN, the only unknown variable in the above equation is $M_{i}$; thus, it can be expressed as a function $f(M_{i})$. Then, we can express $M_{i}$ via its inverse function as follows:

(15) $$M_{i}=f^{-1}\left(n_{idle}\right).$$

At the end of each frame, the ACN can determine how many minislots are in the idle and successful states, allowing it to estimate how many pieces of SUE will have data packets to send in the next frame:

(16) $$M_{i+1}=\left(M_{i}-M_{succ}\right)+\left(M-M_{i}+M_{succ}\right)\left(1-e^{-N_{C}T_{C}\lambda_{i}}\right),$$

where $M_{succ}$ is the number of pieces of SUE that successfully gained access in frame *i*. Since the competition access information contains identity information, $M_{succ}$ is also known to the ACN. The above equation is composed of two parts: one part corresponds to the SUE that did not send packets due to a probability of $1-P_{i}$ and need to retransmit them, and the other part corresponds to those that have just reached the need to access. $\lambda_{i}$ can be iteratively estimated as

(17) $$\lambda_{i}=-\alpha \frac{\ln\left(\frac{M_{i}-M}{M_{i-1}-M-M_{succ}^{\prime }}\right)}{N_{C}T_{C}}+\left(1-\alpha \right)\lambda_{i-1},\;s.t.\;0<a<1.$$

Since the arrival rate does not change drastically in a short time, a portion of the previous frame’s arrival rate is retained. $M_{succ}^{\prime }$ is the number of pieces of SUE that successfully gained access in the previous frame. The ACN can then set the access probability $P_{i+1}$ for the next frame based on the predicted $M_{i+1}$ to ensure that $G=G^{*}$, thereby maximizing the throughput per unit time.

### 4.3. STCA with Variable Frame Length

**Figure 7:**
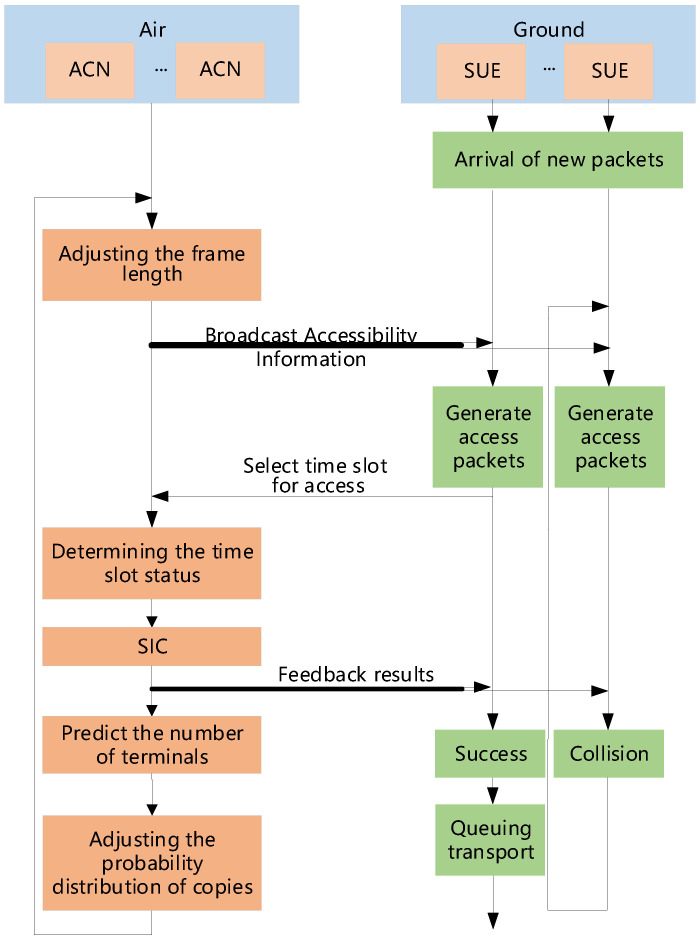
System flowchart of STCA with variable frame length.

The present section describes the flow of STCA with the variable frame length algorithm, and Figure 7 shows the flowchart of the system.

During initial system startup, the satellite equally allocates sub-channel quantities to each aerial access control node. The aerial node receiving this channel resource allocation will broadcast the service transmission channel indices, contention access channel indices, and the current frame’s accessible micro-timeslot count $K_{i}$ to its controlled piece of SUE. Upon receiving these indices, the piece of SUE randomly generates multiple copies, selects multiple timeslots from the access frame, and transmits these copies to the aerial access node.

The aerial control node then performs Successive Interference Cancellation, calculates the number of potentially accessible pieces of SUE for the next frame based on the current copy probability distribution, adjusts the access frame length, and feeds back the access results along with the next frame’s accessible micro-timeslot count to the piece of SUE. SUE that successfully gained access transmits data to the satellite via service transmission channels according to the feedback, while failed SUE awaits the next frame of the contention access channel to reattempt access.

The ACN is configured with $K_{i}$ micro-timeslots in the *i*-th frame. For SUE, there are $M_{i}$ pieces of SUE with data packets to transmit in the *i*-th frame. For a single micro-timeslot, the probability that only one piece of SUE accesses the slot is expressed as

(18) Pidle=Mi∑l=1NCrlKi−1lKilMi.

The estimated number of successfully accessed minislots for the aerial access control node in the *i*-th frame can be obtained by multiplying the above result by $K_{i}$, as follows:

(19) $$n_{\mathrm{idle}}=K_{i}P_{\mathrm{idle}}=f\left(M_{i}\right).$$

To compute $M_{i}$, we need to use the inverse function of the above equation, which is given by

(20) $$M_{i}=f^{-1}\left(n_{\mathrm{idle}}\right).$$

An ACN can determine the status of each minislot at the end of every frame, thereby enabling it to estimate the number of pieces of SUE that will have data packets to transmit in the next frame:

(21) $$M_{i+1}=\left(M_{i}-M_{\mathrm{succ}}\right)+\left(M-M_{i}+M_{\mathrm{succ}}\right)\left(1-e^{-K_{i}T_{C}\lambda_{i}}\right).$$

The $\lambda_{i}$ values are all estimated from the previous frame and then used in the current frame’s calculations. The $\lambda_{i}$ for the current frame can be estimated iteratively using the following formula:

(22) $$\lambda_{i}=-\alpha \frac{\ln\left(\frac{M_{i}-M}{M_{i-1}-M-M_{\mathrm{succ}}^{\prime }}\right)}{K_{i}T_{C}}+\left(1-\alpha \right)\lambda_{i-1}.$$

## 5. Numerical Results

### 5.1. Simulation Setup

To evaluate the performance of the proposed protocols, this section compares the performance of the two protocols proposed in this paper with the Slotted ALOHA protocol and the IRSA protocol. The program running environment is Python 3.11.5.

The CAS slot lasts 0.2 ms, and the TTS slot lasts 4 ms. The system runtime is set to 20,000 ms, which is the length of one superframe. The number of pieces of SUE is 220, and the number of ACNs is 1. The traffic load refers to the average number of packets arriving for all pieces of SUE within 4 ms. The polynomial probability distribution of copies is derived from the effective distributions validated in [7]. In their research, a series of optimal probability distributions were obtained using differential evolution algorithms, which balance the trade-off between computational complexity and system throughput. For our simulation, we select a distribution with moderate complexity (avoiding excessive copy numbers that increase the decoding burden) and proven performance: $\Lambda \left(x\right)=0.5x^{2}+0.28x^{3}+0.22x^{8}$. $N_{C}$ is 200, and $G^{*}$ is 0.7. The initial access probability $P_{1}$ is 1, the initial estimated number of users $M_{1}$ is 220, and the initial estimated arrival rate $\lambda_{1}$ is 1/22.

In the simulation, channel utilization is used as the metric to measure the efficiency of the protocol. The channel utilization *U* is calculated as $U=\frac{N_{TS}}{N_{T}}$, where $N_{T}$ is the total number of TTS slots and $N_{TS}$ is the number of TTS slots with traffic transmission.

The specific parameters of the simulation process are shown in Table 1.

### 5.2. Simulation Results

**Figure 8:**
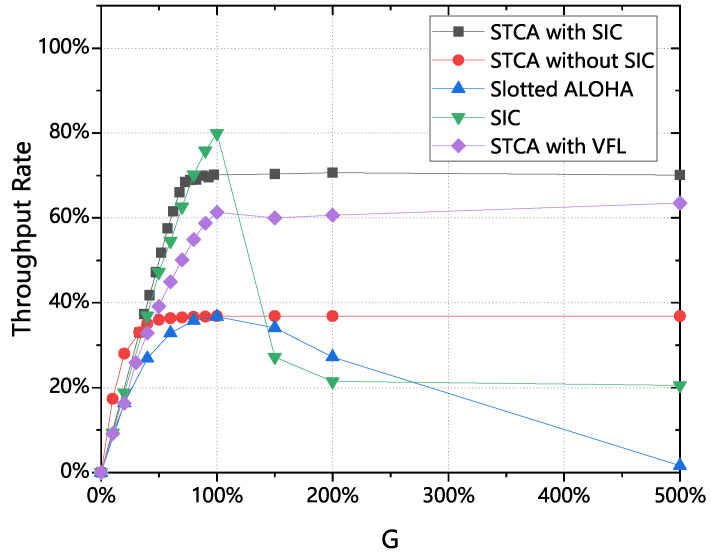
Throughput rate comparison.

**Figure 9:**
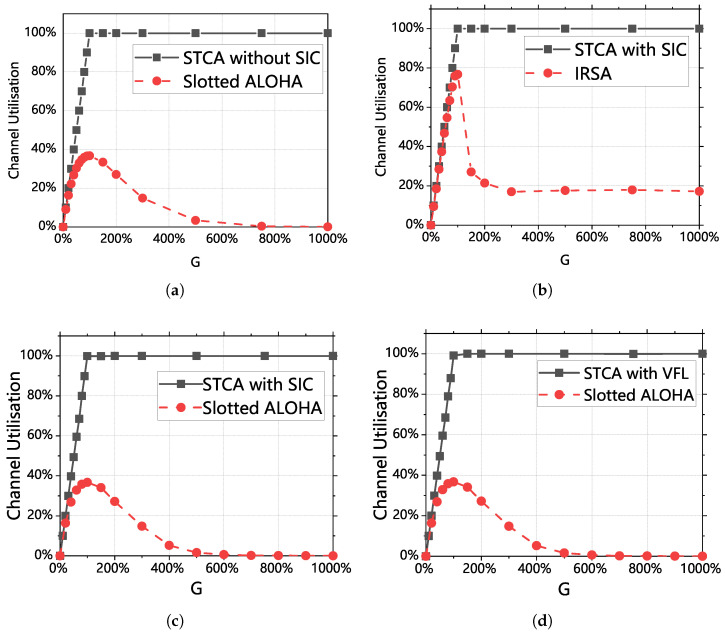
(**a**) Channel utilization comparison between STCA without SIC and Slotted ALOHA. (**b**) Channel utilization comparison between STCA with SIC and IRSA. (**c**) Channel utilization comparison between STCA with SIC and Slotted ALOHA. (**d**) Channel utilization comparison between STCA with VFL and Slotted ALOHA.

Figure 8 shows the throughput rates of the five classes of algorithms (in Figure 8 and Figure 9, VFL denotes variable frame length), and it can be clearly seen that the three classes of algorithms in this section have stable and high throughput rates when the arrival rate is greater than 100%, which is much greater than that of the IRSA algorithm and the Slotted ALOHA algorithm. The throughput of the algorithms with SIC is also better than that of the algorithms without SIC, and by introducing the serial interference technique, this section succeeds in increasing the original maximum throughput rate from about 37% to 70%.

Meanwhile, it can be seen that the stability of STCA with variable frame length can be guaranteed, and different algorithms can be selected according to the different capabilities of equipment: if more attention is paid to fairness and the performance of the air access control equipment is better, then STCA with variable frame length can be selected; if it is only hoped to maximize the utilization of the channel, then STCA with SIC can be selected; if the performance of the air access control equipment is more general, then STCA without SIC can be selected. The three algorithms achieve the desired goal of maximizing the service channel utilization of the satellite, although they are implemented in different ways.

Figure 9 presents a comparison of the TTC channel utilization for four different algorithms. The traffic load of 1000% refers to a scenario where the number of arriving packets is 10 times the maximum capacity of the TTS channel, used to verify the robustness of the proposed STCA protocol under extreme overload conditions. In Figure 9a, neither of the algorithms uses the SIC algorithm, while in Figure 9b, both algorithms implement SIC. In Figure 9c,d, two algorithms that implement SIC are compared with the commonly used algorithms. As shown, both algorithms achieve the theoretical results derived in the analysis. When the traffic load is less than 100%, TTC channel utilization grows almost linearly with the traffic load. This indicates that when the traffic load is below 100%, the ground terminals are able to promptly send their access requests to the satellite, and during the 20 s simulation, nearly all of the traffic data reach the receiving satellite. Under the same conditions, if a Slotted ALOHA algorithm is used, a large number of data packets remain in a retransmission state. Similarly, if IRSA is used, as shown in Figure 9b, a large number of data packets are still in a retransmission state when the traffic load is between 80% and 100%. Specifically, when the traffic load is 100%, about 40% of the data packets fail to reach the satellite terminal.

When the traffic load exceeds 100%, it can be observed from both figures that TTC channel utilization approaches 100%, indicating that the system efficiently utilizes the limited satellite communication channel while minimizing the delay for the ground terminals. Under the same conditions, with IRSA, TTC channel utilization remains almost constant at 20%. In high-traffic-load scenarios, about 80% of the channel resources are wasted, which is unacceptable. This observation underscores the efficiency of the two algorithms designed in this study.

**Figure 10:**
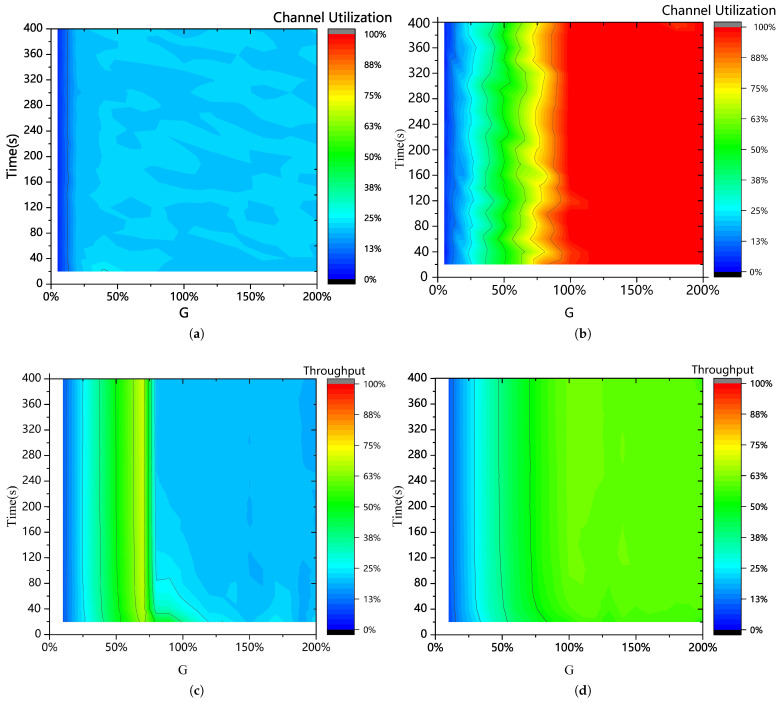
(**a**) Channel utilization of Slotted ALOHA algorithm. (**b**) Channel utilization of STCA algorithm with SIC. (**c**) Throughput of IRSA algorithm. (**d**) Throughput of STCA algorithm without SIC.

To assess the system’s stability, this section evaluates the TTC channel utilization over varying system operational times. Figure 10a shows the channel utilization of the Slotted ALOHA algorithm, while Figure 10b presents the channel utilization of the STCA algorithm without SIC. The channel utilization of the STCA algorithm with SIC is shown in Figure 9, which is consistent with the results of the STCA algorithm without SIC and, therefore, is not repeated here.

It is evident that the relatively simple Slotted ALOHA algorithm produces relatively stable experimental results. Similarly, the STCA algorithm proposed in this paper also exhibits stable results, with its channel utilization significantly outperforming that of the Slotted ALOHA algorithm. Figure 10c illustrates the throughput of the IRSA algorithm, while Figure 10d shows the throughput of the STCA algorithm without SIC. The results indicate that the proposed STCA algorithm demonstrates superior stability compared with IRSA, with throughput remaining consistently high without any decline.

Channel utilization (Figure 10a,b) reflects the efficiency of TTS slot usage, while throughput (Figure 10c,d) measures the rate of successful data access. For STCA algorithms, high throughput directly translates into sustained high channel utilization. Compared with IRSA (Figure 10c), which shows fluctuating throughput under varying loads, STCA without SIC (Figure 10d) maintains consistently high throughput, validating its superior stability.

As shown in Figure 10, the channel utilization of the proposed STCA with SIC (Figure 10b) fluctuates slightly during system operation, while SA (Figure 10a) exhibits milder fluctuations. This is because the higher complexity of STCA with SIC increases the difficulty of prediction and estimation. However, its higher channel utilization makes such minor stability issues negligible.

## 6. Conclusions

This paper proposes a Separated Transmission and Control ALOHA (STCA) protocol, which enhances network performance by introducing airborne access control nodes (ACNs) and separating transmission and control functions. Simulation results show that STCA outperforms existing protocols in key metrics.

STCA with SIC achieves a throughput of 70%, significantly higher than the 37% of Slotted ALOHA. Under high traffic loads (>100%), STCA’s TTC channel utilization approaches 100%, while IRSA’s remains at 20%. Under 100% load, IRSA results in 40% packet loss, whereas STCA ensures that nearly all traffic reaches the satellite. Additionally, STCA solves IRSA’s issue of long frame times by using minislots, optimizing efficiency. These three STCA variants (without SIC, with SIC, and with variable frame length) all maximize satellite channel utilization, offering flexible options for different device capabilities.

In the future, we will extend the STCA protocol to multi-ACN scenarios, focusing on exploring inter-ACN coordination mechanisms to address issues like load imbalance and spectrum interference between adjacent ACNs. Additionally, we will validate the protocol in more realistic contexts by incorporating real-world satellite communication impairments such as channel fading and rain attenuation into the simulation model, aiming to enhance the robustness of STCA in actual deployments.

## Figures and Tables

**Table 1:** Simulation parameter design.

Parameter	Value
Number of ACNs	1
Number of pieces of SUE	220
Frame duration	44 ms
Slot duration	4 ms
Minislot duration	0.2 ms
Data packet duration	4 ms
Access packet duration	0.2 ms
2-copy probability	0.5
3-copy probability	0.28
8-copy probability	0.22
Initial estimated SUE arrival rate	1/22
Initial estimated access probability	1
Maximum iteration count	200
Simulation duration	20,000 ms

## Data Availability

All data generated or analyzed during this study are included in this article.

## Abbreviations

The following abbreviations are used in this manuscript:

IRSA	Irregular Repetition Slotted ALOHA
STCA	Separated Transmission and Control ALOHA
CSMA	Carrier Sense Multiple Access
SA	Slotted ALOHA
DSA	Diversity Slotted ALOHA
CRDSA	Contention Resolution Diversity Slotted ALOHA
CAS	Contention Access Spectrum
RAS	Resource Allocation Spectrum
TTS	Traffic Transmission Spectrum
SIC	Successive Interference Cancellation
VFL	Variable Frame Length
